# Better Safeguards Needed

**Published:** 1897-09

**Authors:** 


### BETTER SAFEGUARDS NEEDED.

The localized outbreak of anthrax at Dubois, Pa., in August, which received considerable attention at the hands of the public press throughout the country, has been investigated and placed under control through the efforts of the Pennsylvania State Veterinary Sanitary Board. This investigation revealed the facts that this outbreak was from an importation of goatskins from China, and that there exist no barriers whatever to prevent these localized outbreaks at any time, inasmuch as no precautionary measures are taken to sterilize these hides or hair (used by plasterers) or any demand made upon the exporters to insure our people from the dangers incidental thereto. Would not the public press do a good work by calling general attention to the importance of this matter? Efforts similar to those so successfully used to prevent the introduction of Asiatic cholera would do much good in guarding our people against this fatal malady—anthrax.

